# Factors associated with non-utilisation of health service for childbirth in Timor-Leste: evidence from the 2009-2010 Demographic and Health Survey

**DOI:** 10.1186/1472-698X-14-14

**Published:** 2014-05-05

**Authors:** Vishnu Khanal, Andy H Lee, Jonia Lourenca Nunes Brites da Cruz, Rajendra Karkee

**Affiliations:** 1 Sanjeevani College of Medical Sciences, Butwal, Rupandehi, Nepal; 2 School of Public Health, Curtin University, Perth, WA, Australia; 3 Department of Pharmacy, Ministry of Health, Dili, Timor Leste; 4 School of Public Health and Community Medicine, BP Koirala Institute of Health Sciences, Dharan, Nepal

**Keywords:** Facility-based childbirth, Home delivery, Maternal health services, Timor-Leste

## Abstract

**Background:**

Timor-Leste is a young developing country in Asia. Most of its infrastructure was destroyed after a long armed conflict for independence. Despite recent expansion of health facilities and investment in healthcare, maternal mortality remains high with most mothers still giving birth at home. This study investigated factors affecting the non-utilisation of health service for childbirth in the aftermath of the independence conflict.

**Methods:**

The Timor-Leste Demographic and Health Survey 2009-2010 was the latest two-stage national survey, which used validated questionnaires to obtain information from 26 clusters derived from 13 districts of the country. Factors influencing non-utilisation of health facility for childbirth were investigated using univariate and multivariable logistic regression analyses, accounting for the cluster sampling and sample weight of the survey.

**Results:**

Of the total 5986 participants included in the study, 4472 (74.8%) did not deliver their last child at a health facility. Lack of education for the mother (adjusted odds ratio (OR): 2.04; 95% confidence interval (CI) 1.56 to 2.66) and her partner (OR: 1.45; 95% CI 1.14 to 1.84), low household wealth status (OR: 5.20; 95% CI 3.93 to 6.90), and rural residence (OR: 2.83; 95% CI 2.22 to 3.66), were associated with increased likelihood of non-utilisation of health facility for childbirth. Working mothers (OR: 1.55; 95% CI 1.32 to 1.81), who had high parity (OR: 1.78; 95% CI 1.36 to 2.32) and did not attend antenatal care service (OR: 4.68; 95% CI 2.65 to 8.28) were also vulnerable for not delivering at a health facility. Conversely, the prevalence of non-utilisation of health facility for childbirth reduced with increasing number of service components received during antenatal care visits (OR: 0.72; 95% CI 0.64 to 0.80).

**Conclusions:**

Only a quarter of Timorese women delivered at a health facility. In order to reduce maternal mortality, future interventions should target disadvantaged mothers from poor families, those residing in rural areas, have higher parity but no education, and who seldom attend antenatal care service, by improving their utilisation of health facility for childbirth.

## Background

Globally 287,000 mothers die per year as a result of pregnancy and childbirth related causes, mostly from developing countries [1]. Many of these maternal deaths are avoidable if women use a health facility for delivery. Provision of skilled care during delivery, with access to emergency obstetrical service, is an effective strategy to reduce maternal mortality [2]. Various factors can influence the decision on facility-based childbirth, including maternal education, wealth status, residential location, distance to health facility, antenatal care, and perceived quality of service [3-5]. In particular, antenatal care provides an opportunity to educate the women, enhances their communication with health workers, and also facilitates them to prepare for childbirth [4]. Similarly, when the quality of antenatal care meets expectation and medical standard, the rate of facility-based childbirth will be high [3,6].

Timor-Leste is a young developing country in Asia. About 70% of its health infrastructure was destroyed during the independence conflict with Indonesia in the late 1990s. The country has a population of 1.07 million [7], with 40% of the people live below the poverty line [7]. The conflict has negatively affected social development and health status. Maternal mortality was estimated to be 500-660 per 100,000 live births [7]. After the conflict, the Timor-Leste government promoted facility delivery and strived to provide basic services through different health facility levels. By 2010, 5 hospitals, 65 community health centres and 211 health posts had been constructed [7]. The Timorese constitution has affirmed that health is a human right and as such healthcare should be provided free of charge through public health facilities [8]. Maternal health (safe motherhood) service was an integral part of this package [9,10], but place of delivery remained not a focus due to the lack of birthing facilities. Later in 2007, along with refurbishment of 200 birthing centres, the government shifted its policy towards facility-based childbirth [10,11]. The 20-year Timor-Leste Strategic Development Plan 2011-2013 [12] targeted to have 65% of deliveries being attended by skilled birth attendants. The plan also committed to improve obstetric service for early detection and management of obstetric complications.

Given that Timor-Leste is a new country, there has been little published information on the utilisation of facility service for childbirth. Knowledge on the underlying factors can assist with the formulation of appropriate policy in safe motherhood programme [13]. This study investigated non-utilisation of health service for childbirth in the aftermath of the conflict using the latest data from the national Demographic and Health Survey (DHS) 2009-2010.

## Methods

This study utilised the dataset from the Timor-Leste DHS 2009-2010 [14]. It was the second demographic and health survey conducted in the country as part of the Global Measure DHS involving more than 50 countries [14]. The *Childrenrecode* data set which included place of delivery within the last five years was used. Of the 9,828 childbirths recorded in the survey, the present study included a total of 5,986 last born children living with the respondents. The DHS, being a two-stage survey, used validated questionnaires to obtain information from 26 clusters within 13 districts [15].

The binary outcome variable was “non-utilisation of health facility for childbirth”, with home delivery coded as 1 and facility delivery coded as 0 [15]. A “health facility” for childbirth can be either public (national hospital, referral hospital, community health centre, health post, SISCa post, and other public hospitals) or private sector facility (private hospital, Marie stopes, NGO run facilities) [3].

Plausible socio-demographic variables were selected based on a review of the relevant DHS literature [3,16,17]. In addition to individual level factors (maternal age, maternal education, paternal education, maternal occupation, birth order, frequency of antenatal care (ANC) visits, ANC service components, intake of iron during pregnancy, pregnancy complication, and age of mother at childbirth), information on household level factors (religion, wealth status) and community level factors (residential location) [18] was also extracted from the DHS database. Frequency of ANC visits was expressed as: 0, 1-3, and ≥ 4 [19]. Number of ANC service components was also considered. There were four components reflecting service quality, namely, weight, blood pressure, urine sample and blood sample being taken [15]. Intake of iron during pregnancy was reported as either yes or no. Pregnancy complication was classified as present if the mother experienced at least one symptom. Wealth index was regrouped into poor (lower 40%), middle (middle 40%) and rich (upper 20%) [20,21]. Both maternal and paternal education were re-categorised as no education, primary, and secondary or higher. Residential location (rural versus urban) was used as a proxy to account for the geographical variations in the country.

The Timor-Leste DHS 2009-2010 was approved by the ethics review board of the ICF Macro International and the Ministry of Health, Timor-Leste [15]. The male and female participants gave their own consent whereas mothers or care takers provided consent for their children and minors.

All statistical analyses were performed using the SPSS package version 20. After summarising the data by descriptive statistics with corresponding 95% confidence intervals, factors associated with facility delivery were examined via chi-square test. The associations were then investigated using univariate and multivariable logistic regression analyses. A hierarchical modelling strategy was adopted [18,22], with individual level factors being assessed first in the multivariable model. Household level factors were then added to the model along with significant individual level factors, followed by ascertaining community level factors at the final step [23]. Collinearity was also assessed and one variable among the correlated set of independent variables was selected for inclusion in the final model. To account for cluster sampling and sample weight of the DHS [24], a complex sample analysis method was deemed appropriate for a more precise estimation of proportion and association [25].

## Results

### Sample characteristics

Table 1 presents the characteristics of participants. Of the 5,986 mothers, the majority (60.3%) were aged between 20 and 34 years and 3/4 (75.3%) of them resided in rural areas. One in every three mothers (32.9%) did not have formal education and over 60% reported not working. While 86.9% of mothers attended at least one ANC visit, only 55% achieved the recommended four visits. With regard to the four ANC service components, although most women were weighted (84.8%) and had their blood pressure measured (81.7%), relatively few women had their urine sample (15.7%) and blood sample (12.2%) taken. Only 7.8% of women received all four components during their ANC visit. A vast majority (93.7%) of women experienced at least one pregnancy related complications (vaginal bleeding, severe lower abdominal pain, severe headache, convulsion, blurred vision and swelling of hands and face).

**Table 1:** Characteristics of sample (N = 5986)

**Factor**	**N**	**Percent**
**Individual level factors**		
**Maternal age** (years)		
≤ 19	191	3.0
20-34	3563	60.3
35-49	2232	36.7
**Maternal education**		
Secondary or higher	2256	39.6
Primary	1714	27.6
No education	2016	32.9
**Paternal education**		
Secondary or higher	2593	44.4
Primary	1685	27.3
No education	1708	28.3
**Maternal occupation**		
Not working	3608	61.4
Working	2378	38.6
**Birth order**		
1	853	14.1
2-3	1680	29.0
≥ 4	3453	56.9
**Frequency of antenatal care visits**		
≥ 4	3341	55.0
1-3	1860	31.9
0	781	13.1
**Antenatal service components**		
0	883	14.6
1	210	3.4
2	3772	62.9
3	667	11.3
4	454	7.8
**Intake of iron during pregnancy**		
Yes	3900	63.1
No	2080	36.9
**Pregnancy complications**		
Present	5610	93.7
Absent	376	6.3
**Age of mother at childbirth**		
≤ 19	462	7.4
20-34	3968	66.7
35-49	1556	25.9
**Household level factors**		
**Religion**		
Roman Catholic	5857	98.0
Others	129	2.0
**Wealth status**		
Rich (upper 20%)	988	20.7
Middle (middle 40%)	2456	39.5
Poor (lower 40%)	2542	39.8
**Community level factors**		
**Residential location**		
Urban	1383	24.7
Rural	4603	75.3

### Factors influencing facility delivery

Of the 5,986 childbirths, 1514 (25.2%) were facility-based, thus giving a non-utilisation of health service prevalence of 74.8%. As shown in Table 2, except religion and presence of pregnancy complications, all other independent variables appeared to be associated with the outcome variable. Table 3 summarises the results of logistic regression analyses. Lack of education for the mother (adjusted odds ratio (OR): 2.04; 95% confidence interval (CI) 1.56 to 2.66) and her partner (OR: 1.45; 95% CI 1.14 to 1.84), low household wealth status (OR: 5.20; 95% CI 3.93 to 6.90), and rural residence (OR: 2.83; 95% CI 2.22 to 3.66), were associated with increased likelihood of non-utilisation of health facility for childbirth. Working mothers (OR: 1.55; 95% CI 1.32 to 1.81), those with high parity (OR: 1.78; 95% CI 1.36 to 2.32) and did not attend ANC service (OR: 4.68; 95% CI 2.65 to 8.28) were also vulnerable for not delivering at a health facility. Conversely, the prevalence of non-utilisation of health facility for childbirth reduced with increasing number of service components received during ANC visits (OR: 0.72; 95% CI 0.64 to 0.80).

**Table 2:** Distribution of variables by delivery location

**Factor**	**Home delivery (N = 4472)**	**Facility delivery(N = 1514)**	P*
	**n (%; 95% CI)**^#^	**n (%; 95% CI)**^#^	
**Individual level factors**			
**Maternal age** (years)			< 0.001
≤ 19	147 (3.0; 2.5, 3.7)	44 (2.6; 1.9, 3.7)	
20-34	2684 (58.1; 56.4, 59.8)	879 (66.9; 63.6, 70.1)	
35-49	1817 (38.9; 37.2, 40.5)	415 (30.5; 27.5, 33.6)	
**Maternal education**			< 0.001
Secondary or higher	1395 (30.1; 28.3, 31.9)	861 (67.7; 64.8, 70.5)	
Primary	1418 (30.1; 28.3, 32.0)	296 (20.0; 17.8, 22.5)	
No education	1835 (39.8; 37.7, 42.1)	181 (12.3, 10.4, 14.4)	
**Paternal education**			< 0.001
Secondary or higher	1710 (36.4; 34.5, 38.3)	883 (68.3; 65.4, 71.1)	
Primary	1398 (29.7; 28.2, 31.2)	287 (19.9; 17.4, 22.7)	
No education	1540 (33.9; 32.1, 35.8)	168 (11.8; 10.1, 13.7)	
**Maternal occupation**			< 0.001
Not working	2652 (57.9; 56.0, 59.7)	956 (72.0; 69.0, 74.8)	
Working	1996 (42.1; 40.3, 44.0)	382 (28.0; 25.2, 31.0)	
**Birth order**			< 0.001
1	571 (12.2; 11.2, 13.3)	282 (19.9; 17.8, 22.1)	
2-3	1241 (26.8; 25.5, 28.2)	493 (35.7; 32.4, 39.1)	
≥ 4	2836 (61.0; 59.3, 62.8)	617 (44.4; 41.2, 47.7)	
**Frequency of antenatal care visits**			< 0.001
≥ 4	2409 (50.5; 48.2, 52.8)	932 (68.2; 64.5, 71.7)	
1-3	1471 (32.4; 30.5, 34.3)	389 (30.6; 27.2, 34.1)	
0	768 (17.1; 15.4, 19.0)	17 (1.2; 0.7, 2.1)	
**Antenatal service components**			< 0.001
0	861 (19.0; 17.2, 21.0)	22 (1.4; 0.9, 2.3)	
1	183 (3.8; 3.2, 4.5)	27 (2.1; 1.4, 3.1)	
2	2914 (61.9; 59.8, 64.0)	858 (65.9; 62.0, 69.5)	
3	419 (9.4; 8.3, 10.5)	248 (17.1; 14.9, 19.5)	
4	271 (5.9; 5.1, 6.9)	183 (13.5; 11.1, 16.4)	
**Intake of iron during pregnancy**			< 0.001
Yes	2914 (59.4; 57.0, 61.7)	986 (74.0; 69.1, 78.3)	
No	1730 (40.6; 38.3, 43.0)	350 (26.0; 21.7, 30.9)	
**Pregnancy complications**			0.131
Present	4340 (93.3; 92.2, 94.3)	1270 (95.0; 92.9, 96.5)	
Absent	308 (6.7; 5.7, 7.8)	68 ( 5.0; 3.5, 7.1)	
**Age of mother at childbirth**			< 0.001
≤ 19	342 (7.2; 6.4, 8.1)	120 (7.8; 6.2, 9.7)	
20-34	3017 (64.9; 63.3, 66.4)	951 (72.3; 69.0, 75.3)	
35-49	1289 (27.9; 26.4, 29.4)	267 (19.9; 17.4, 22.8)	
**Household level factors**			
**Religion**			0.290
Roman Catholic	4550 (98.1; 97.5, 98.6)	1307 (97.5; 95.8, 98.5)	
Others	98 (1.9; 1.4, 2.5)	31 (2.5; 1.5, 4.2)	
**Wealth status**			< 0.001
Rich (upper 20%)	406 (10.2; 8.7, 11.9)	582 (51.9; 47.3, 56.4)	
Middle (middle 40%)	1907 (40.9; 38.7,43.1)	549 (35.2; 31.4, 39.3)	
Poor (lower 40%)	2335 (48.9; 46.4, 51.4)	207 (12.9; 10.9, 15.2)	
**Community level factors**			
**Residential location**			< 0.001
Urban	740 (14.4; 12.8, 16.2)	643 (55.3; 50.8, 59.6)	
Rural	3908 (85.6; 83.8, 87.2)	695 (44.7; 40.4, 49.2)	

*Chi-square test of association; ^#^Adjusted for clustering and sampling weight.

**Table 3:** Factors associated with non-utilisation of health facility for childbirth from hierarchical logistic regression analysis

**Factor**	**Crude odds ratio (95% CI)**	**Adjusted odds ratio (95% CI)**	**P**
**Maternal education**			< 0.001
Secondary or higher	1.00	1.00	
Primary	3.38 (2.84, 4.04)	1.47 (1.18, 1.85)	
No education	7.33 (5.97, 9.01)	2.04 (1.56, 2.66)	
**Paternal education**			0.010
Secondary or higher	1.00	1.00	
Primary	2.79 (2.34, 3.34)	1.22 (0.97, 1.53)	
No education	5.41 (4.47, 6.55)	1.45 (1.14, 1.84)	
**Maternal occupation**			< 0.001
Not working	1.00	1.00	
Working	1.87 (1.61, 2.17)	1.55 (1.32, 1.81)	
**Birth order**			< 0.001
1	1.00	1.00	
2-3	1.23 (0.99, 1.51)	1.47 (1.15, 1.87)	
≥ 4	2.24 (1.88, 2.68)	1.78 (1.36, 2.32)	
**Frequency of antenatal care visits**			< 0.001
≥ 4	1.00	1.00	
1-3	1.43 (1.19, 1.72)	1.13 (0.93, 1.37)	
0	18.85 (10.82, 32. 83)	4.68 (2.65, 8.28)	
**Antenatal service components**	0.52 (0.48, 0.57)	0.72 (0.64, 0.80)	< 0.001
**Wealth status**			< 0.001
Rich (upper 20%)	1.00	1.00	
Middle (middle 40%)	5.89 (4.73, 7.34)	2.69 (2.14, 3.37)	
Poor (lower 40%)	19.28 (15.01, 24.76)	5.20 (3.93, 6.90)	
**Residential location**			< 0.001
Urban	1.00	1.00	
Rural	7.33 (5.76, 9.34)	2.83 (2.22, 3.66)	

## Discussion

This study found three quarters of the Timorese mothers did not utilise health facility for childbirth. There are several reasons to explain the low utilisation of facility delivery service. When the country gained its independence status in 2002, more than 70% of the health infrastructure had been destroyed by the militia [11]. A previous study of maternity waiting homes reported that in the early years after independence, the country had to relied on home delivery because suitable and accessible health facilities were lacking [10]. Unavailability of skilled personnel may also contribute to the low facility delivery rate. Furthermore, similar to other less developed countries in Asia, childbirth at home is often regarded as a natural phenomenon and traditional culture, with the women either ignoring or unaware of the risk associated with home delivery [2].

ANC is the first step of the continuum of service provided during pregnancy [26]. It has been documented that ANC can influence the use of subsequent services after the pregnancy stage [26]. This study found that non-attendance of ANC was positively associated with non-utilisation of health facility for childbirth, whereas receiving more components of ANC service had the opposite effect. ANC has a number of benefits. It provides an opportunity for communication between mothers and health workers [27,28]. The WHO has recommended that health workers should help mothers to prepare a plan for childbirth [26,29]. As a result of such preparation during ANC, mothers are likely to attend health facility for childbirth [4,17,30]. In case of any health problem or pregnancy related issue, it can be rectified during the pregnancy stage [17,30]. The provision of additional services further increases their likelihood for facility-based childbirth [4]. Several studies have also reported the positive effects of having ANC and better quality of ANC on facility delivery [17,28,30].

Women residing in rural areas were almost three times more likely not to utilise health facility for childbirth than their urban counterparts. The rural-urban disparities in health status have been well documented [31]. Those living in urban areas typically have better transportation, economic status and access to hospital and health services, which enhance their opportunity for facility delivery. Similarly, significant association was evident between lower wealth status and non-utilisation of facility for childbirth. It is known that poor household members are unlikely to use health services [31,32]. Poor women have less economic power to pay for service or commodity they need, and are thus disadvantaged and less empowered than their rich counterparts [31]. In many developing countries, the poorest sections are also excluded from the mainstream society which ultimately lead to under-utilisation of skilled care during delivery [31].

The finding regarding education is supported by the literature [17,32]. Educated couples usually have better jobs or income and a higher empowerment level. Education also enhances communication skills to negotiate for better pregnancy care and place for childbirth [17,31]. Women with education are also more confident in decision making on delivery location than their uneducated peers [31]. They are able to understand the information provided by health workers, which is crucial for following instructions related to pregnancy and planning for childbirth [31].

The relationship between birth order and non-utilisation of health service for childbirth is consistent with studies in other countries [16,17,33]. Unlike first time mothers, higher parity women have experience of childbirth, and are less likely to seek assistance if no complications arise [16]. They perceive childbirth as a natural process and may feel confident to give birth at home [16]. Moreover, it has been reported that if mothers had encountered bad experience with health workers in previous childbirths, they may prefer home delivery instead [17].

Finally, maternal occupation was another influencing factor. A review has concluded that maternal working may decrease the likelihood of facility delivery if such paid occupation is poverty induced [2]. Similar findings have also been reported in other developing countries [34,35]. In Timor-Leste, the majority of its population lives in rural areas under low socio-economic conditions [36]. The working status of women is mainly determined by poverty level, with non-working mothers being more able to afford to deliver at a health facility.

A major strength of the present study is the use of the latest national dataset while accounting for the survey sampling method, so that the results are generalizable to the whole population. Although the information collected was retrospective in nature, childbirth is a vital event for mothers and other family members. Therefore, misclassification of the place of delivery was unlikely. In view of our findings, future studies should focus on the feasibility of implementing incentive schemes and safe motherhood programmes, similar to those in other developing countries [3,37]. This study highlights the positive influence of ANC visits during pregnancy. Recently, the Timor-Leste government introduced the Servisu Integrado Sude Communita (SISCa) program (integrated health service program in the community) which mobilises community health volunteers to encourage pregnant women to attend ANC services, and in turn increases their utilisation of health facility for childbirth [38]. The inequalities reported between poor and rich households, and between rural and urban areas, should be addressed. Increasing 24-hour birthing centres in rural areas, and providing incentives for transportation may be viable solutions to close the gap. In addition, the reasons and attitudes underlying the utilisation of delivery service should be explored, including cultural preference and belief, decision making process and perceived quality of care among mothers.

## Conclusions

Three quarters of Timorese mothers did not deliver at a health facility. Women with higher parity, residing in rural areas, from poor household, with no education, working and not attending ANC, were more likely to incur non-facility based childbirth. Future intervention to improve maternal health and promote health facility delivery should focus on these groups of disadvantaged mothers.

## Competing interests

No competing interests declared for all authors.

## Authors’ contributions

VK formulated the study concept, performed statistical analysis and drafted the manuscript. AHL supervised the project, contributed to data analysis and revision. JDC and RK contributed to literature review and interpretation of findings. All authors have read and approved the final version for publication.

## Pre-publication history

The pre-publication history for this paper can be accessed here:

http://www.biomedcentral.com/1472-698X/14/14/prepub
